# PDA Closure Rates and Safety after Repeated Courses of Cyclooxygenase Inhibitors in Preterm Infants: A Systematic Review and Meta-Analysis

**DOI:** 10.64898/2026.09.09.26360534

**Published:** 2026-09-13

**Authors:** Junichi Saito, Yuka Sano Wada, Kota Yoneda, Kunio Ogawa, Nobuhiko Kan, Tetsuya Isayama, Katsuaki Toyoshima

## Abstract

**Background:**

Cyclooxygenase (COX) inhibitors have been used to treat hemodynamically significant patent ductus arteriosus (PDA) in preterm infants. If the PDA remains hemodynamically significant after an initial course, additional courses of COX inhibitors or surgical intervention are considered. This systematic review evaluates PDA closure rates and safety after repeated courses of COX inhibitors in preterm infants.

**Methods:**

Embase, Medline, and two other databases were searched through April 2026 (pre-registered in PROSPERO [CRD42023454003]). Studies reporting PDA closure rates after two or more courses of COX inhibitors were included. Risk of bias was assessed using Joanna Briggs Institute tools. Meta-analyses with random-effects models were performed to calculate the proportions of PDA closure. Multilevel mixed-effects meta-regression was used to compare closure rates across treatment courses while accounting for within-study correlations.

**Results:**

A total of 33 studies were included. The PDA closure rates after the first, second, and third courses of COX inhibitors were 62% (95% confidence interval [CI] 57–67%), 46% (95% CI 40–52%; *p* < 0.001 vs the first course), and 38% (95% CI 27–51%; *p* = 0.001 vs the first course), respectively. Lower closure rates with repeated courses were also observed across most subgroups (extremely preterm/extremely low birth weight, ibuprofen, indomethacin, and intravenous administration), except for the second course of oral administration. Data on adverse events were limited, preventing meta-analysis.

**Conclusions:**

PDA closure rates after the second and third courses of COX inhibitors were lower than those after the first course in preterm infants. Evidence regarding the safety of repeated courses remains limited.

**Impact:**

- This systematic review is the first study to integrate the PDA closure rates in preterm infants following the second and third courses of COX inhibitor treatment.
- The PDA closure rate after the first course of COX inhibitor treatment was 62%, whereas those after the second and third courses were 46% and 38%, respectively.
- Multilevel meta-regression and pairwise comparisons showed that later COX inhibitor courses yielded significantly lower PDA closure rates than the first.
- Evidence with adverse events after repeated COX inhibitor treatment was limited and required further investigation.

## Full Text Availability

The license terms selected by the author(s) for this preprint version do not permit archiving in PMC. The full text is available from the preprint server.

